# Preclinical Evaluation of a Phage T4‐Based Multi‐Epitope Nanoparticle Vaccine Against Porcine Epidemic Diarrhea Virus

**DOI:** 10.1155/tbed/8438783

**Published:** 2026-06-14

**Authors:** GuoQing Zhao, ShiDan Zhang, YuMin Zhang, ShengJing Jiao, XiaoYan Zeng, LuRu Zhao, Jie Wang, JingJiao Ma, YuQiang Cheng, HengAn Wang, YaXian Yan, JianHe Sun, ZhaoFei Wang

**Affiliations:** ^1^ Shanghai Key Laboratory of Veterinary Biotechnology, School of Agriculture and Biology, Shanghai Jiao Tong University, Shanghai, 201100, China, sjtu.edu.cn

**Keywords:** epitopes, phage, porcine epidemic diarrhea virus, vaccine

## Abstract

Porcine epidemic diarrhea virus (PEDV) continues to threaten the global swine industry, and currently available vaccines often provide incomplete protection against emerging virulent strains. In this study, we developed a phage T4‐based multi‐epitope nanoparticle vaccine targeting PEDV. B and T cell epitopes derived from the PEDV spike (S) protein were identified through immunoinformatic analyses and assembled into a tandem epitope construct (Pep) using flexible linkers. In silico analyses predicted favorable antigenic properties of Pep and a stable interaction with Toll‐like receptor 3 (TLR3). The recombinant Pep protein was expressed in *Escherichia coli* and subsequently displayed on T4 phage to generate the T4‐Pep vaccine. Characterization revealed that each phage carried approximately 800 copies of Pep. In mice, immunization with T4‐Pep induced significantly higher PEDV‐specific IgG levels and virus‐neutralizing antibody titers than immunization with the Pep. Following PEDV challenge, T4‐Pep–immunized mice showed improved protection and reduced intestinal damage, as confirmed by histopathological analysis. Together, these findings highlight the potential of phage T4 as an efficient and immunogenic platform for delivering viral epitopes, offering a promising strategy for the development of vaccines against PEDV and related pathogens.

## 1. Introduction

Porcine epidemic diarrhea virus (PEDV) is chiefly transmitted via the fecal‐oral route and primarily infects the animal’s intestines [1]. Suckling piglets are especially vulnerable to PEDV infection. Following infection, mortality rates range from 50% to 100% [2, 3]. The virus exhibits a marked tropism for intestinal epithelial cells, inducing severe villous atrophy that culminates in malabsorptive diarrhea, dehydration, and ultimately, death [4]. Although conventional vaccines are used to prevent PEDV, they are not very effective against emerging variant strains [5, 6].

Following vaccination, cells trigger a complex series of immune responses [7]. Virus‐infected cells are primarily eliminated through Cytotoxic T lymphocytes (CTL)‐mediated immune responses [8]. Concurrently, helper T‐cell epitopes (HTL) facilitate B cell activation and differentiation, leading to the production of antibodies [9]. Employing immunoinformatic tools to predict epitopes for B and T lymphocytes has significantly speeded up the design and development of antiviral vaccines. Currently, research into vaccines for PEDV encompasses a variety of technical approaches. These include live‐attenuated and inactivated PEDV vaccines [10]; an oral PEDV vaccine based on cationic liposomes [11]; a subunit vaccine [12]; nanoparticle vaccines [13]; and mRNA PEDV vaccines encapsulated in lipid nanoparticles [14], to name a few. However, PEDV has evolved and mutated due to external pressures, making it increasingly difficult to prevent and control [6, 15]. The PEDV virus is still tricky to contain successfully [16, 17]. Currently, epitope‐based vaccines have emerged as a promising class of immunogens, particularly within veterinary medicine [5]. Subunit vaccines incorporating defined viral epitopes offer a compelling alternative to traditional approaches, combining enhanced safety profiles with the capacity for precise immune targeting. The spike (S) protein of PEDV, which facilitates viral attachment and membrane fusion, represents the primary target for neutralizing antibodies and encompasses multiple immunodominant B‐ and T‐cell epitopes essential for protective immunity [18, 19]. However, peptide‐based antigens alone are typically poorly immunogenic and necessitate efficient delivery systems or immunostimulatory adjuvants to provoke adequate responses.

The T4 bacteriophage has recently been used to develop vaccines by displaying antigens on its capsid surface [20, 21]. T4 bacteriophage has emerged as a versatile and highly immunogenic vaccine platform. Its surface architecture features two dispensable capsid proteins—Soc and Hoc—which permit the display of heterologous peptides through either genetic fusion or chemical conjugation [22]. Studies have shown that the T4 phage vector can enhance the immune response independently [23]. Furthermore, the repetitive and symmetrical arrangement of T4 phage capsid proteins resembles the pathogen‐associated molecular patterns (PAMPs) of human viruses that activate pattern recognition receptors (PRRs). These features likely bind to extracellular PRRs, triggering the transmission of inflammatory signals [24]. Additionally, the large quantity of the major T4 phage capsid protein enables antigens to be incorporated into the phage in a concentrated manner. This multivalent presentation strongly activates B‐cell receptors, thereby inducing a robust humoral immune response. This property may also optimize immunogenicity by regulating the antigen copy number and spacing [25]. By leveraging the innate immunostimulatory properties of bacteriophages, phage‐based vaccine platforms circumvent many limitations associated with conventional vaccine approaches, enhancing the immunogenicity of target antigens [22, 26]. In addition, phages themselves can induce immune responses [27]. Recent progress in phage‐based delivery systems has opened new avenues for commercial vaccine development [28]. Collectively, these advances support the exploration of novel phage‐vectored vaccines against PEDV as a promising strategy to mitigate outbreaks.

There is accumulating evidence that the T4 bacteriophage has great potential as a vaccine platform against viruses [21, 26]. This research utilized bioinformatics to identify epitopes within the S protein of PEDV. These epitopes were then assembled in tandem and displayed on T4 phages using an in vitro assembly method. Subsequent evaluation of their properties and immunogenicity suggests that this construct holds promise as a vaccine candidate, providing both a theoretical and practical basis for PEDV prevention and control.

## 2. Materials and Methods

### 2.1. Prediction of Epitopes

The PEDV S protein (GenBank: AVK78141.1) served as the template for epitope prediction. In parallel, we obtained the S protein sequences of various PEDV genotypes from the NCBI database and performed an alignment analysis using ESPript 3.2 [29].

The ABCpred [30] was employed to identify B‐cell epitopes (BCL) of 16 residues and a threshold of 0.85, yielding 29 candidate epitopes in total. Next, we used ElliPro [31] to predict conformational epitopes, setting the screening parameters to a minimum score of 0.5 and a maximum distance of 6 Å. This ultimately yielded 28 candidate epitopes. The overlapping sequences from the ABCpred and ElliPro predictions were then identified and integrated based on the epitope length. For fully overlapping epitopes, the sequence predicted by ABCpred was adopted. For partially overlapping epitopes, the two sequence segments were fuzed to form a complete epitope covering both regions. Subsequently, we used VaxiJen [32] to analyze the antigenicity and ToxinPred2 [33] to evaluate their toxicity. Epitopes with a predicted antigenicity score exceeding 0.5 and lacking toxicity were selected for downstream applications.

Owing to the scarcity of relevant data for epitope prediction, we leveraged the presumed similarity between the anchor residues of human HLA and porcine MHC class II haplotypes. Accordingly, NetMHCpanII‐4.1 [34] was employed to predict HTL epitopes (H2‐IAb, length(s): 15). Subsequently, VaxiJen was used to filter for antigenic candidates, selecting those with a score exceeding 0.4. For the prediction of CTL epitopes, the NetMHCpan‐4.1 tool [34] was applied to assess peptide binding to multiple SLA class I alleles, with the epitope length fixed at nine amino acids. The resulting candidates were further evaluated for antigenicity via VaxiJen, where epitopes scoring above 1.2 were prioritized.

### 2.2. Vaccine Development and Evaluation

The epitopes were concatenated using AAY, EAAAK, KK, and GPGPG as linkers. Subsequently, the CDCRGDCFC peptide was conjugated to the epitope string via the flexible EAAAK linker, resulting in a protein designated as Pep. A three‐dimensional structural model of Pep was then predicted in silico using AlphaFold2 [35].

Using ChimeraX [36], we mapped the selected dominant epitopes onto the S protein structure (PDB ID: 6U7K) to visualize their distribution.

We performed molecular docking of Pep (PDB ID: 7C77) with Toll‐like receptor 3 (TLR3) using ClusPro [37], with the resulting interactions visualized via LigPlot+. Subsequently, the immunogenicity of Pep was simulated using C‐ImmSim [38]. Molecular dynamics (MD) simulations of the complex were performed using iMODS [39].

### 2.3. Plasmid Construction

Previously established methods [21] were followed for constructing the pRbSoc plasmid. After codon optimization of the designed vaccine Pep, *Xho*I and *Hin*dIII restriction sites were inserted at either end of the gene. The gene fragment (Table 1) was then synthesized by Sangon Biotech and cloned into the pET‐28a and pRbSoc plasmids, resulting in the construction of the pET‐Pep and pRbSoc‐Pep plasmids.

**Table 1:** RB69 Soc sequences.

Name	Nucleotide sequences (5′ to 3′)
RB69 Soc	AAGCTTACCACTTACTGGTGTAGGGGTAAACATTGCAGCGTTCTTAGTACGCCAATCTGCTGCGTCAGTCACCACAGTAGTGTAAGCAGCTTTTTCAGATGGGAAAGTCTGATAATGAGCATCAGCAATCCGATGTTCGTTTGAATAAATCTCAAACGGTACAGAAACTTCCATACCTTTAACTTCTTTACCTTCACCAGCAGGATGCGTAAAGGTTTTGATGTTTACATAACCACCGGATCC
Pep	AAGCTTTGCGACTGCCGTGGTGACTGCTTCTGCGAAGCTGCTGCTAAAGAACACTCTGTTGTTGGTATCACCTGGGAAGCTGCTGCTAAATCTATGTCTATCCGTACCGAATACCTGGAAGCTGCTGCTAAATCTAAATTCAACGTTCAGGCTCCGGCTGTTGTTGTTCTGGGTGGTGGTCCGGGTCCGGGTAAATTCAACGTTCAGGCTCCGGCTGTTGTTGTTCTGGGTGGTTACGGTCCGGGTCCGGGTCTGGGTGTTTCTGTTTACGACCCGGCTTCTGGTCGTGTTGTTCAGAAAAAAAAAGACTGGTCTCGTGTTGCTACCAAATGCTACAACTCTGGTGGTTGCGCTGCTGCTTACAACGTTACCAACTCTTACGGTTACGTTTACAAATCTCAGGACTCTAACGGTCCGGGTCCGGGTCCGTCTTTCAACGACCACTCTTTCGTTAACATCACCGTTTCTGCTTCTTTCGGTGGTCACTCTGGTGCTAACCTGATCGCTCTCGAG

*Note:* The underlines indicate the introduced restriction enzyme sites of XhoⅠ and Hind Ⅲ.

### 2.4. Expression and Purification of Proteins

Following previously established methods [21], protein expression was induced with IPTG for 7 h at 37°C.

### 2.5. Western Blotting

Following electrophoretic separation, the proteins were electrotransferred onto a PVDF membrane. We blocked the membrane overnight at 4°C in 5% (w/v) skim milk in TBST (prepared by adding 0.1% Tween‐20 to Tris‐buffered saline) and then washed it with TBST. The membrane was then treated with 1:5000 diluted PEDV‐positive mouse serum for 1 h at 37°C. Subsequently, the membrane was rinsed thrice using TBST buffer, applied 1:5000‐diluted HRP‐conjugated anti‐mouse IgG (GAM‐HRP) (Abcam, UK), and conducted incubation with mild shaking for 1 h.

### 2.6. Preparation and Purification of T4 Soc^−^ Phage

T4 phage was prepared by infecting logarithmic‐phase *E. coli* P301 at an MOI of 0.1 and incubating overnight. The culture was centrifuged at 35,000 *g* for 35 min, then resuspended it in 10 mL of phosphate‐buffered saline (PBS) containing 600 μL of chloroform (shook the suspension at 4°C for 35 min). A subsequent centrifugation step at 5600 *g* for 18 min was performed to remove chloroform, yielding the purified phage preparation.

### 2.7. In Vitro Assembly of T4‐Pep Vaccine

Soc‐Pep and T4 Soc^−^ were conjugated using the previously described method [21]. To maximize the Pep display, T4 Soc^−^ and Soc‐Pep were mixed at various molar ratios and incubated at 4°C for 55 min. The sample was subjected to centrifugation at 35,000 *g* for 40 min to remove any free Soc‐Pep. Next, the T4‐Pep phage was resuspend in PBS at a pH of 7.4. The number of copies was calculated using the previously described method [21].

### 2.8. Mouse Immunizations and Challenges

Female mice of the BALB/C were organized into four groups, as shown in Table 2, and received oral vaccinations at weeks 0, 2, 4, and 6. The T4‐Pep and Pep groups were inoculated with an equal amount of Pep protein, respectively. The Pep concentration in the T4‐Pep group was calculated using the following formula: the product of 50 μg and Avogadro’s constant, divided by the molecular weight of Pep, and finally divided by the number of copies. Mouse serum was collected 14 days after each immunization. On day 15 after the third boost, all mice were orally inoculated with 100 TCID_50_ of the PEDV‐CHN/SH‐2012‐5/2012 virus (the mice’s weight was recorded daily). CHN/SH‐2012‐5/2012 was maintained in our laboratory.

**Table 2:** Grouping for mouse immunization.

Group	Dose (μL)
PBS (*n* = 9)	100
T4 (*n* = 9)	100
T4 Pep (*n* = 9)	100
Pep (*n* = 9)	100

### 2.9. Specific Antibody Detection

The PEDV antigen originates from a strain of PEDV‐CHN/SH‐2012‐5/2012 preserved in our laboratory. Plates coated overnight (4°C) with 100 ng/well PEDV were blocked with 2.5% skim milk (2 h, 37°C). After PBST washes, serially diluted sera (1:200–1:51,200) were added. Then, GAM‐HRP (1:5000) was applied and incubated under the same conditions for an additional hour. Following six PBST washes, TMB substrate (Solarbio, Beijing, China) was applied for 15 min at 37°C. The reaction was stopped with 5% H_2_SO_4_ and the OD_450_ was measured.

### 2.10. Serum Neutralization Assay

Serum neutralizing antibodies against PEDV were determined by a virus neutralization assay. After heat‐inactivating (56°C, 30 min) the collected serum, a two‐fold serial dilution was performed and incubated with 100 TCID_50_ of PEDV at 37°C for 1 h. The samples were loaded into 96‐well plates preseeded with Vero cell monolayers, followed by 1 h incubation at 37°C, and the supernatant was discarded. The cells were maintained in Dulbecco’s Modified Eagle Medium (serum‐free) and monitored daily for the development of cytopathic effects (CPEs).

### 2.11. Hematoxylin and eosin (HE) Staining

Proximal jejunal segments collected at 7 days post‐challenge, approximately 4–5 cm distal to the pylorus, were fixed in 4% paraformaldehyde for 48 h. Gradient ethanol dehydration, paraffin embedding, and sectioning were performed on collected tissues, followed by routine HE staining.

### 2.12. Quantitative Real‐Time Reverse Transcription PCR (qRT‐PCR)

Viral load in the proximal jejunal tissue was determined by qRT‐PCR. Total RNA was extracted from approximately 1 g of jejunal tissue using an RNA extraction kit (Accurate Biology, China). Reverse transcription was conducted to generate the cDNA. For amplification, we employed primers targeting the PEDV N gene (F: 5′‐AAAGGAAATAAGGACCAGCA‐3′ and R: 5′^′^‐AAATGCCAATTGGAAGGTTG‐3′^′^). The N‐protein cDNA sequence was cloned into PUC19 to create the PUC19‐N plasmid. After determining the concentration with a Nano‐100 Micro‐Spectrophotometer (Beyotime, China), the copy number concentration was calculated based on the molecular weight and Avogadro’s constant. Then, a 10‐fold gradient dilution (10^2^–10^8^ copies/μL) was prepared to create a standard curve. Standard curves were used to measure the absolute copy number of viral RNA.

### 2.13. Statistical Analysis

GraphPad Prism 8.0.2 was adopted for statistical analysis, with all data displayed as mean ± SD.

## 3. Results

### 3.1. Epitope Vaccine Design for PEDV

**Figure 1:**
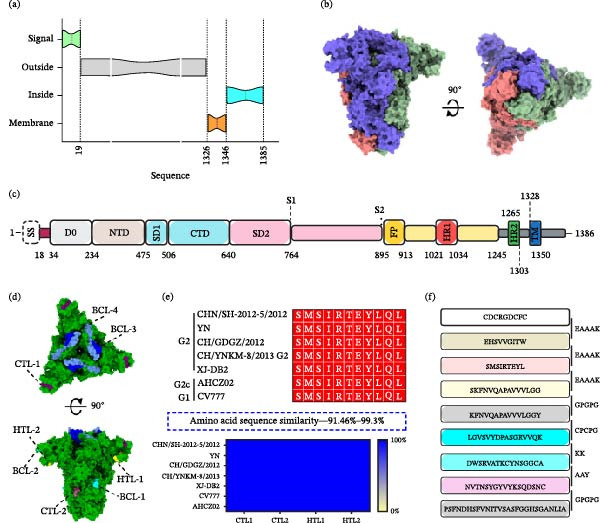
In silico analysis and epitope screening of the PEDV S protein for vaccine design. (a) Predicted transmembrane topology of the PEDV S protein. (b) Three‐dimensional structure of the S protein (PDB ID: 6U7K) used as a template for conformational epitope prediction. (c) Schematic domain architecture of the PEDV S protein, illustrating the boundaries of the S1 and S2 subunits and the extracellular localization of the 19–1326 region. (d) Surface distribution of the identified dominant epitopes on the S protein trimer (colored regions), with most epitopes mapping to the S1 domain. (e) Sequence conservation analysis of linear epitopes across representative PEDV strains. Multiple sequence alignment of S proteins, including the highly pathogenic G2c strain (AHCZO2), revealed high epitope conservation. (f) Different epitope sequences were linked together using peptide linkers.

The PEDV S protein was selected as the target antigen for epitope discovery because it is the principal surface glycoprotein involved in host‐cell entry [40]. Transmembrane‐topology analysis showed that amino acids 19–1326 lack transmembrane segments, indicating that this region is suitable for epitope screening (Figure 1a).To improve screening reliability, linear and conformational epitopes were predicted using multiple bioinformatics tools, including ABCpred, ElliPro, NetMHCIIpan‐4.1 and NetMHCpan‐4.1. The S protein structure (Figure 1b) used for conformational epitope prediction was retrieved from the PDB (PDB ID: 6U7K). As shown in Table 3, none of these screened epitopes were found to be toxic. The outer surface of the S protein structure is where these epitopes are exposed, according to spatial analysis (Figure 1d). The majority of these epitopes are located within the S1 region (Figure 1c and Table 3). To assess epitope conservation, we retrieved S protein sequences from various PEDV strains in the NCBI database, including the highly pathogenic G2c (AHCZO2) [6]. Sequence alignment revealed over 91% similarity among the analyzed strains, with the identified linear epitopes being highly conserved across them (Figure 1e). These epitopes were conjugated in a specific sequence using EAAAK, KK, AAY, and GPGPG linkers. In addition, we added a peptide segment (CDCRGDCFC), which is capable of binding to αvβ3 integrin [41], to the C‐terminus. This should enhance the vaccine’s targeting ability.

**Table 3:** Candidate epitopes.

Type	Name	Epitope	Antigenicity	Toxic	Position
BCL	BCL1	LGVSVYDPASGRVVQK	0.5620 > 0.5	Nontoxic	878–893
BCL	BCL2	DWSRVATKCYNSGGCA	0.9787 > 0.5	Nontoxic	188–203
BCL	BCL3	NVTNSYGYVYKSQDSN	0.5069 > 0.5	Nontoxic	556–571
BCL	BCL4	PSFNDHSFVNITVSASFGGHSGANLIA	0.6834 > 0.5	Nontoxic	505–531
CTL	CTL1	EHSVVGITW	1.5570 > 1.2	Nontoxic	160–168
CTL	CTL2	SMSIRTEYL	1.3770 > 1.2	Nontoxic	789–792
HTL	HTL1	SKFNVQAPAVVVLGG	0.4039 > 0.4	Nontoxic	37–51
HTL	HTL2	KFNVQAPAVVVLGGY	0.4278 > 0.4	Nontoxic	38–52

### 3.2. Candidate Vaccine Modeling and TLR3 Molecular Docking

**Figure 2:**
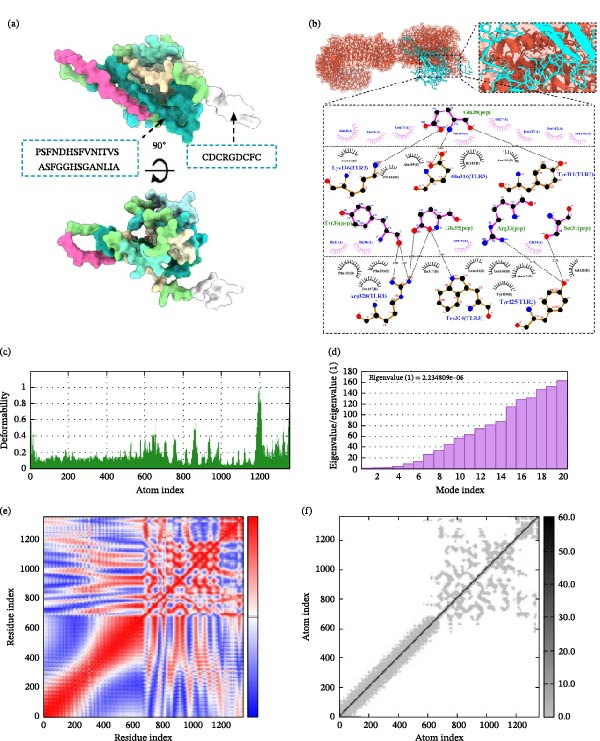
In silico characterization and interaction analysis of the multi‐epitope vaccine construct Pep with TLR3. (a) Structure of the multi‐epitope vaccine candidate Pep, generated using AlphaFold 2. (b) Protein–protein docking analysis of the Pep‐TLR3 complex performed with the ClusPro server and visualized using LigPlot+. (c) Deformability analysis of the Pep‐TLR3 complex obtained from iMODS molecular dynamics simulations. (d) Eigenvalue plot showing the calculated eigenvalue of 2.234809 × 10^−6^, reflecting the high rigidity and global stability of the complex. (e) Covariance matrix illustrating the coupling patterns between residue pairs, where red, white, and blue represent correlated, uncorrelated, and anticorrelated motions, respectively. (f) Elastic network representation of the Pep‐TLR3 complex. Dense gray regions at the binding interface indicate strong interatomic interactions.

Following sequential linkage of the selected epitopes, the resulting multi‐epitope construct was termed Pep. Its three‐dimensional conformation was predicted via AlphaFold 2, as illustrated in Figure 2a. To this end, the vaccine candidate must effectively engage immune receptors on target cells. Given that TLR3 targeting agents have been proven to boost vaccine potency by compensating for insufficient interferon secretion and protecting against highly pathogenic PEDV infection [42], we evaluated the interaction between our designed Pep and TLR3. Pep and TLR3 were designated as ligand and receptor, respectively. The resulting docked complex was analyzed using LigPlot+, as shown in Figure 2b. Under the predicted binding mode, Pep formed stable interactions with TLR3, with key hydrogen bonds involving Pep residues Glu38, Tyr36, Glu35, Ser31, and Arg33. To further assess the dynamic stability of the Pep‐TLR3 complex, MD simulations were conducted using iMODS. Deformability analysis (Figure 2c) revealed that most peaks were below 0.5, indicating that the backbone residues in this region are resistant to deformation and contribute to a compact structural conformation. The calculated eigenvalue for the complex was 2.234809 × 10^−6^ (Figure 2d), reflecting high rigidity and global stability. The covariance matrix (Figure 2e) illustrated the coupling patterns between residue pairs. Additionally, the elastic network of the complex (Figure 2f) showed dense gray regions at the binding interface, indicating strong interatomic interactions (stiffer springs). Collectively, these results demonstrate that the Pep‐TLR3 complex adopts a stable binding conformation with limited fluctuations, supporting its potential to effectively engage the immune receptor.

### 3.3. In Silico Assessment of Immune Activation Effects

**Figure 3:**
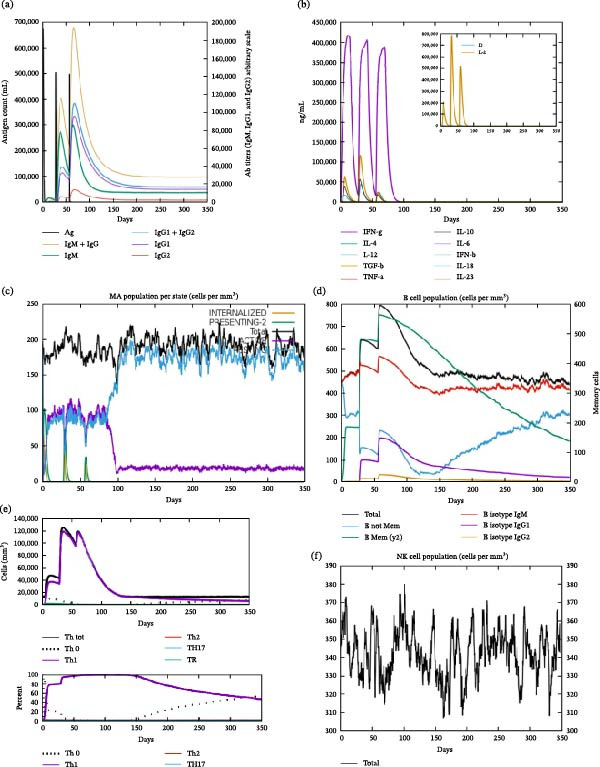
C‐ImmSim‐based immunogenicity prediction of the Pep vaccine candidate. (a) Immunoglobulin profiles showing IgM and IgG antibody levels following three rounds of antigenic stimulation. (b) Cytokine and interleukin concentrations throughout the simulated immune response. (c) Macrophage dynamics, indicating activation upon antigen exposure. (d) B cell population dynamics, including total and memory B cells, reflecting a robust humoral response. (e) T cell population dynamics, depicting the expansion of helper and cytotoxic T lymphocytes. (f) NK cell dynamics, showing increased activity poststimulation.

C‐ImmSim was used to simulate the immune response induced by the Pep construct after three rounds of antigen stimulation. The results demonstrated that following three rounds of antigenic stimulation, Pep effectively activated multiple immune cell populations, including B cells (Figure 3d), natural killer (NK) cells (Figure 3f), macrophages (Figure 3c), and T cells (Figure 3e). Notably, high levels of IgG antibodies were induced (Figure 3a). Meanwhile, the concentrations of various cytokines remained consistently high (Figure 3b). These findings suggest that Pep possesses strong immunogenic properties.

The epitope conservation analysis, structural protection prediction, and molecular docking results described above are intended solely to furnish a theoretical rationale and structural reference for the design of the multivalent vaccine molecules in this study. They do not constitute direct validation of the molecules’ biological immunogenicity. Further confirmation of the relevant immunogenicity and protective efficacy is required through subsequent experiments.

### 3.4. Construction of T4‐Pep Vaccine

**Figure 4:**
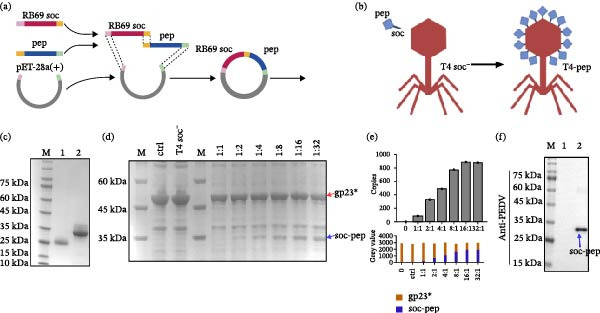
Construction and characterization of the T4‐Pep nanoparticle vaccine. (a) Schematic diagram of the Soc‐Pep expression vector construction. (b) Illustration of the phage‐based antigen display strategy: the Soc‐Pep fusion protein is anchored onto Soc‐deficient T4 phage capsids through Soc–T4 Soc^−^ interactions. (c) SDS‐PAGE analysis of purified Soc‐Pep and Pep proteins following expression in *E. coli* and affinity purification (M: marker, 1: Pep, 2: Soc‐Pep). (d) Coupling efficiency of T4 Soc^−^ phage (5 × 10^10^ PFU) with Soc‐Pep at various molar ratios of Pep to Soc binding sites (1:1–32:1). A control reaction using Pep alone at a 10:1 molar ratio (ctrl) confirmed the specificity of Soc‐mediated binding. (e) Grayscale quantification of gp23 ^∗^ and Soc‐Pep bands and calculation of the average Pep copy number displayed per T4 phage. (f) Western blot analysis confirming specific binding of Soc‐Pep to PEDV‐positive mouse serum (M: marker, 1: T4, 2: Soc‐Pep).

Since RB69 Soc protein enables efficient display of exogenous peptides at both terminals [23], this system was adopted to construct a T4 phage vaccine against PEDV. A fusion protein, Soc‐Pep, was generated by linking Pep to RB69 Soc, enabling Soc to anchor Pep onto the T4 phage capsid via Soc–T4 Soc^−^ interactions (Figure 4b). The expression vector for Soc‐Pep was constructed using the strategy depicted in Figure 4a. High yields of Soc‐Pep were obtained in *E. coli*, and the proteins were purified (Figure 4c). To optimize antigen loading, T4 Soc^−^ (5 × 10^10^ PFU) and Soc‐Pep were mixed at different molar ratios ranging from 1:1 to 32:1 (Figure 4d). Image Pro Plus 6.0 grayscale analysis of protein bands was applied to quantify surface‐displayed Pep levels. As expected, Pep alone failed to bind T4 Soc^−^ even at a 10:1 ratio (ctrl lane, Figure 4d), confirming that binding is strictly Soc‐dependent. Display levels increased with the input ratio and reached saturation at approximately 16:1 (Figure 4e). Based on these results, recombinant T4‐Pep was constructed. The Pep copy number per phage was calculated using the formula: copies = (Soc‐Pep grayscale value) × 49 × 930/(33 × (gp23 ^∗^ grayscale value)), yielding an average of approximately 850 copies per phage (Figure 4e). Western blotting further confirmed that T4‐Pep was specifically recognized by PEDV‐positive mouse serum (Figure 4f).

### 3.5. T4‐Pep Induces Robust Specific Antibody Production

**Figure 5:**
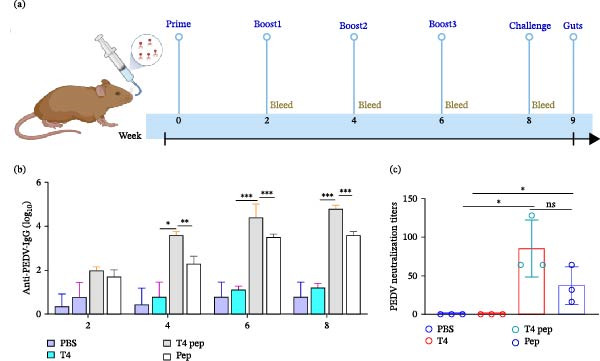
Immunogenicity evaluation of the T4‐Pep vaccine in mice. (a) Schematic diagram of the immunization schedule. Mice were immunized via oral gavage at weeks 0, 2, 4, and 6, with serum collected at weeks 2, 4, 6, and 8 postimmunization. (b) PEDV specific IgG antibody titers in serum samples collected at weeks 2, 4, 6, and 8 postimmunization, measured by ELISA (*n* = 3, all data were analyzed using a two‐tailed *t*‐test). (c) Neutralizing antibody titers in week 8 serum samples determined by a fixed virus dilution method. Serial dilutions of immune mouse serum were incubated with PEDV, and neutralization titers were calculated as the reciprocal of the highest dilution showing complete neutralization (*n* = 3, all data were analyzed using a two‐tailed *t*‐test). (ns, not significant;  ^∗^ *p* < 0.05;  ^∗∗^ *p* < 0.01;  ^∗∗∗^ *p* < 0.001).

To evaluate the immunogenicity of the T4‐Pep vaccine, mice were immunized via oral gavage with 1.2 × 10^12^ PFU T4‐Pep phage (equivalent to 50 μg of Pep), 50 μg of free Pep, T4, and PBS as a negative control (Table 2). The immunization regimen followed the schedule outlined in Figure 5a. Serum was collected and PEDV‐specific antibody titers were quantified by ELISA. Mice immunized with free Pep produced only low levels of PEDV‐specific IgG compared with those immunized with T4‐Pep, which induced a significantly stronger antibody response (Figure 5b). Notably, the T4‐Pep formulation induced high‐titer specific IgG antibodies, reaching a peak titer of 5 × 10^4^. At week 8, neutralizing antibody titers in the serum were assessed using a fixed virus dilution method. The T4‐Pep‐immunized group demonstrated higher neutralizing activity against live PEDV compared to the Pep‐alone group (Figure 5c). Collectively, these findings demonstrate that the T4 phage nanoparticle platform exerts potent immunostimulatory effects.

### 3.6. T4‐Pep Provided Protection Against PEDV Challenge

**Figure 6:**
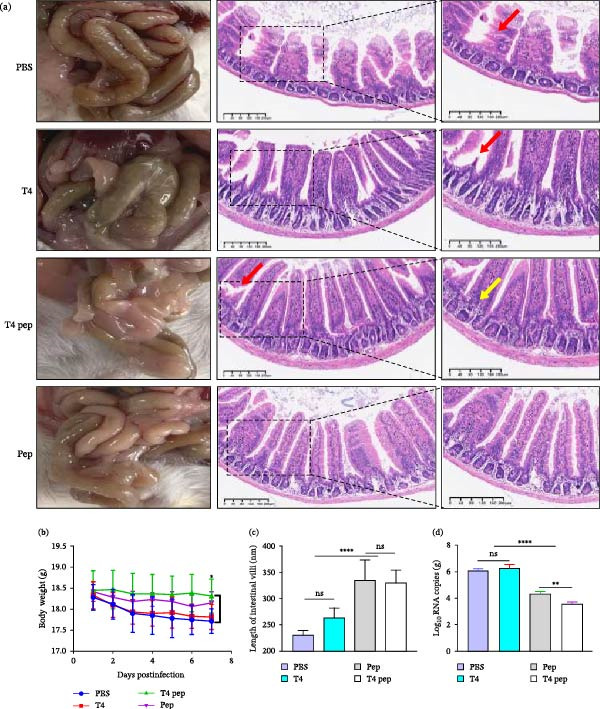
Protective efficacy of Pep T4 against PEDV challenge in mice. (a) Representative histological images of mouse intestinal tissue (scale bar: 200 μm). Pathological alterations include villous injury (red arrows) and crypt hyperplasia (yellow arrows). (b) Daily body weight changes in mice over 7 days post‐PEDV infection (*n* = 6, all data were analyzed using a two‐tailed *t*‐test). (c) Villus length measurements in the proximal jejunum (*n* = 6, all data were analyzed using a two‐tailed *t*‐test). (d) Viral loads in the proximal jejunum of challenged and control mice, quantified by qRT‐PCR. (*n* = 3, all data were analyzed using a two‐tailed *t*‐test). (ns, not significant;  ^∗^ *p* < 0.05;  ^∗∗^ *p* < 0.01;  ^∗∗∗^ *p* < 0.001;  ^∗∗∗∗^ *p* < 0.0001).

Subsequent to the completion of booster immunization, PEDV strain CHN/SH‐2012‐5/2012 was used to infect mice so as to explore the in vivo defense effect of T4‐Pep. Their body weight changes were then monitored for seven consecutive days. As shown in Figure 6b, mice in theT4 and PBS groups exhibited progressive weight loss following viral challenge, whereas those immunized with T4‐Pep maintained relatively stable body weights throughout the monitoring period. On day 7 postinfection, intestinal tissue samples were collected for histopathological evaluation. HE staining revealed that the T4‐Pep‐immunized group displayed intact small intestinal villi with no significant pathological alterations, in contrast to the Pep, T4, and PBS‐immunized groups (Figure 6a). Specifically, the T4‐ and PBS‐immunized groups showed marked villus atrophy and damage (red arrows, Figure 6a, c), while the Pep‐only group exhibited mild villus injury and crypt hyperplasia (yellow arrows, Figure 6a, c). Analysis of viral load in intestinal tissues revealed that the viral load in the tissues of mice vaccinated with the T4‐Pep vaccine was significantly lower than in those immunized with the Pep vaccine (Figure 6d). Collectively, these results demonstrate that the T4 phage nanoparticle platform confers robust protection against PEDV infection in mice, effectively reducing viral replication and preserving intestinal integrity.

## 4. Discussion

PEDV still poses a severe menace to the pig breeding industry worldwide, inflicting substantial economic losses across all age groups of pigs [43]. In China, recent large‐scale outbreaks of diarrhea have been reported in pig populations, with PEDV implicated as the primary viral etiology in 50.90% of cases and detected in 66.67% of affected farms [17]. Although commercial vaccines against PEDV are currently available, the continuous genetic evolution of circulating strains has compromised their effectiveness, highlighting the urgent need for novel vaccine platforms capable of conferring broad and durable protection [44]. In this study, we designed a multi‐epitope peptide derived from the PEDV S protein and constructed the T4‐Pep candidate vaccine using bacteriophage T4 nanoparticles as a delivery platform. This approach integrates immunoinformatic prediction, structural biology analysis, and nanotechnology to address the inherent limitations of low immunogenicity in traditional subunit vaccines and to assess the potential of oral immunization in inducing immune responses.

Vaccination initiates a complex immune response. During this procedure, B cells mediate humoral immunity via antibody responses, while CTLs and HTLs are responsible for orchestrating cellular immunity, which is essential for clearing viruses [45, 46]. The identification of viral epitopes capable of engaging these distinct lymphocyte populations is therefore fundamental to rational vaccine design. Immunoinformatic approaches have emerged as powerful tools for predicting protective antigenic epitopes and accelerating vaccine development [47, 48]. The S protein was used as the target for screening vaccine epitopes in this study. Our analysis indicates that the region spanning amino acids 19–1326 is located in the extracellular domain. Further sequence alignment of S proteins from multiple PEDV strains, including the highly pathogenic G2c (AHCZO2) strain, revealed over 91.46% similarity, and the identified linear epitopes exhibited high conservation across these strains. This significant conservation suggests that vaccines based on these epitopes may provide cross‐protection against multiple PEDV variants, which is a key characteristic for addressing the ongoing circulation of emerging strains such as G2b and G2c.

In this study, we employed immunoinformatic analysis to predict and screen immunodominant epitopes derived from the PEDV S protein, building upon established epitope prediction frameworks [21]. Beyond epitope selection, effective vaccine design must balance immunogenicity enhancement with acceptable reactogenicity and safety profiles [7]. To further assess vaccine‐receptor interactions, we performed molecular docking between the multi‐epitope construct (Pep) and TLR3 using the Cluspro server. TLR3 recognizes double‐stranded RNA intermediates generated during PEDV replication, thereby initiating host innate immune and interferon responses [49]. Nevertheless, upon cell infection, PEDV employs multiple viral proteins to inhibit the TLR3 signaling pathway and evade host immune surveillance [50]. Therefore, targeting TLR3 represents a key strategy to antagonize PEDV immune evasion and restore host antiviral defenses. Molecular docking can predict the binding affinity of vaccine peptides to TLR3, verifying whether the designed vaccine can effectively bind TLR3 at the structural level. Additionally, C‐ImmSim simulations were used to predict that the Pep construct would induce robust humoral and cellular immune responses. Despite their promise, epitope‐based vaccines face inherent limitations related to the weak immunogenicity of peptide antigens, necessitating efficient delivery platforms to potentiate immune activation. Recent advances in nanobiotechnology have reframed viruses not merely as pathogens but as functional nanoparticles with diverse biomedical applications, including vaccine delivery. Structural proteins from viruses including adeno‐associated virus (AAVs) [51] and bacteriophages [28] have been successfully engineered to produce vaccines. Of these platforms, bacteriophage T4 shows the most promise as a vaccine delivery system.

In recent years, researchers have extensively explored novel strategies, such as subunit vaccines [12], viral vector vaccines [52], nucleic acid vaccines [53], and mucosal delivery systems [11], in an attempt to overcome the limitations of traditional vaccines when it comes to preventing and controlling PEDV. However, most of these novel vaccines still face challenges such as insufficient immunogenicity and limited antigen presentation efficiency [2]. Compared to other PEDV vaccine candidates, the T4‐Pep platform exhibits distinct immunological advantages. One recent investigation developed recombinant *Lactobacillus paracasei* to produce subunit vaccine candidates against PEDV [14]. Following oral immunization of pregnant mice with this recombinant strain, a gradual increase in PEDV‐specific antibody titers was observed in both serum and intestinal tissues over time. However, despite a statistically significant elevation compared to the control group, the overall antibody levels remained relatively modest. These findings suggest that immunization with the S1 protein alone may possess limited intrinsic immunogenicity, underscoring the potential benefit of multivalent display platforms such as T4‐Pep in enhancing humoral immune responses. Results demonstrated that equivalent Pep content delivered via the T4 carrier evoked higher serum IgG levels than separate Pep vaccination. These results indicate that the T4 phage delivery platform developed in this study specifically addresses these critical shortcomings in PEDV vaccine development. It demonstrates significant advantages in terms of antigen presentation efficiency and other aspects, providing a practical and feasible technical pathway for the development of novel vaccines that are better suited to the characteristics of PEDV infection and offer more stable protective effects.

Although the candidate vaccine developed in this study shows great potential for application, several aspects warrant further investigation. First, this study used mice as an animal model rather than pigs, the natural host; therefore, the characteristics of the induced immune response and the actual protective efficacy still need to be further validated and evaluated in a porcine model. Second, this study did not conduct direct measurements of mucosal‐related indicators, making it difficult to fully reflect the body’s mucosal immune status. Future studies could incorporate mucosal site‐specific detection indicators to more systematically evaluate the vaccine’s immunogenicity. Furthermore, as this vaccine was designed based on antigenic epitopes rather than the full‐length antigen, the immune profile it induces differs somewhat from that of whole‐antigen immunization, and the underlying protective mechanisms require further elucidation.

In conclusion, this study involved the development of a PEDV vaccine platform using the T4 bacteriophage as a vector and further assessed its ability to elicit immune reactions. In murine models, T4‐Pep exerted prominent protective effects against PEDV infection, hence serving as a highly prospective candidate vaccine for PEDV prevention.

## Author Contributions

**GuoQing Zhao**: conceptualization, validation, methodology, writing – original draft. **ShiDan Zhang**: methodology, validation, writing – review and editing. **YuMin Zhang and ShengJing Jiao**: validation. **XiaoYan Zeng**: formal analysis. **LuRu Zhao**: methodology. **JingJiao Ma**: investigation. **YuQiang Cheng**: visualization. **HengAn Wang**: supervision. **YaXian Yan and JianHe Sun**: funding acquisition. **ZhaoFei Wang:** project administration, conceptualization.

## Funding

This research was funded by the Science and Technology Commission of Shanghai Municipality (Grant 23N51900300) and the National Natural Science Foundation of China (Grant 32373097).

## Disclosure

All participants in this research have approved the finalized article.

## Ethics Statement

The experimental procedures involving mice strictly adhered to the Guiding Principles in the Care and Use of Animals (China) and received approval by the Ethical Committee for Animal Experiments of Shanghai Jiao Tong University (China) (Approval Number 202201312).

## Conflicts of Interest

The authors declare no conflicts of interest.

## Data Availability

The data that support the findings of this study are available upon request from the corresponding author. The data are not publicly available due to privacy or ethical restrictions.
